# Factors associated with depression and anxiety in the adult population of Qatar after the first COVID-19 wave: a cross-sectional study

**DOI:** 10.1007/s44202-021-00009-z

**Published:** 2021-12-20

**Authors:** Salma Mawfek Khaled, Iman Amro, Lina Bader, Peter Woodruff, Majid A. Alabdulla, Tarek Bellaj, Yousri Marzouk, Youssef Hasan, Ibrahim M. Al-Kaabi, Peter M. Haddad

**Affiliations:** 1 Social and Economic Survey Research Institute –SESRI, Qatar University, P.O. Box: 2713, Doha, Qatar; 2 Department of Public Health, College of Health Sciences, Qatar University, P.O. Box 2713, Doha, Qatar; 3 Department of Population Medicine, College of Medicine, Qatar University, P.O. Box 2713, Doha, Qatar; 4 University of Sheffield, Western Bank, Sheffield, S10 2TN UK; 5 Hamad Medical Corporation, P.O. Box 3050, Doha, Qatar; 6 College of Art and Sciences, Qatar University, P.O. Box: 2713, Doha, Qatar; 7 Clinical Science Department, College of Medicine, Qatar University, P.O. Box 2713, Doha, Qatar; 8 Division of Psychology and Mental Health, University of Manchester, Manchester, UK

**Keywords:** Depression, Anxiety, Associated factors, COVID-19 pandemic, Qatar

## Abstract

There is limited data from Arabic-speaking countries on risk factors for depression and anxiety during the COVID-19 pandemic. Country-specific data is necessary given differences in culture, demographics, and COVID-19 infection and mortality rates. The main purpose of the study is to identify the factors associated with symptoms of depression-anxiety in the adult population of Qatar during the first year of the COVID-19 pandemic. We conducted a cross-sectional online survey in Qatar between July and December 2020 after Qatar’s first COVID-19 wave and before the beginning of the second wave. Depression-anxiety was defined as a cut-off of 20 or higher on the Patient Health Questionnaire-Anxiety Depression Scale (PHQ-ADS). Of 1138 participants, 71.0% were female, 69.0% Arabs, 70.0% Non-Qataris, and 77.0% were < 40 years (the median age in Qatar is 32 years). In a fully-adjusted model, six variables were significantly associated with moderate-to-severe levels of depression or anxiety on the PHQ-ADS; Arab ethnicity (OR = 1.67, p = 0.026), never married versus married (OR = 1.69, p = 0.015), prior history of psychiatric disorder versus no history (OR = 1.80, p = 0.009), increased worries due to social media use for COVID-related news/updates (OR = 1.72, p = 0.003), a history of COVID-19 (OR = 1.76, p = 0.039), loneliness (OR = 1.91, p < 0.001), and lower levels of religiosity (OR = 0.96, p = 0.039). These associations also pertained in the reduced model, with the exception of religiosity which was only marginally statistically significant (OR = 0.97, p = 0.055). The potential risk factors identified may assist with anxiety and depression prevention in future COVID-19 waves, and similar national events, and assist with early intervention to treat sufferers.

**Supplementary Information:**

The online version contains supplementary material available at 10.1007/s44202-021-00009-z.

## Background

The first cases of coronavirus disease 2019 (COVID-19), caused by Severe Acute Respiratory Syndrome Coronavirus 2 (SARS-CoV-2), were reported in Wuhan, China, in December 2019. In March 2020 WHO declared the outbreak a pandemic, with 177 million confirmed cases and over 3.8 million fatalities worldwide by June 2021 [1]. Depression and anxiety are major public health problems due to their high prevalence, occurrence during working life and relatively low rates of treatment, all of which lead to a high disability. Hence, the pandemic may increase burden of anxiety and depressive disorders through fear of illness, isolation, bereavement, unemployment, and financial insecurity.

Rates of depression and anxiety in the US and Europe increased in the early months of the pandemic [2, 3]. In the UK, mental health deteriorated in April 2020, one month after the first national lockdown [4]. The deterioration persisted and by October 2020 had not return to pre-pandemic levels [5]. A 20-week longitudinal study spanning the first lockdown in England showed the highest levels of depression and anxiety in the early stages [6]. Both studies [5, 6] showed mental health improvements, with trajectories differing between individuals, although 10% of subjects had deteriorating or consistently poor mental health throughout 6-month period.

Qatar, on the west coast of the Arabian Peninsula, has a population of 2.9 million, median age of 32 years, and 90% of whom are expatriate workers [7]. To date, there have been no data published on the mental health of the general adult population in Qatar during the pandemic. A study conducted in the general populations of seven Arab countries (Egypt, Kuwait, Jordan, Saudi Arabia, Algeria, Iraq, and Palestine) in the early pandemic reported that 17.0% experienced moderate or severe depressive symptoms measured by the PHQ-9, while 6.3% experienced moderate or severe symptoms of generalized anxiety disorder on the GAD-7 [8]. Using the same measures, a large survey (N= 4132) conducted online in Kuwait during the final five days of the first national lockdown (25th to 30th of May, 2020) reported 30.1% depression and 25.3% anxiety [9]. An online community adult survey from the United Arab Emirates (UAE) conducted between April 8th and April 22nd, 2020 (N = 1,039) reported 55.7% depression and 58.4% anxiety using the same cut-offs (score of ≥10) for these scales.

Most existing studies of the mental health impact of COVID-19 pandemic in the Arabic-speaking region are cross-sectional studies that have used convenience samples. The study we report also adopts this methodology. Such studies cannot determine how the prevalence of psychiatric morbidity during the pandemic compares to the pre-pandemic situation. However, this methodology does allow the identification of sociocultural factors associated with psychological distress, which may help in prevention and early intervention for mental illness in current and future pandemics. The significant social differences to western countries including younger population, high proportion of migrants relative to nationals, high level of communal religious observance, and multigenerational living arrangements with large household sizes emphasize the importance of studies in Arabic countries. These factors have been impacted by the isolation and social distancing measures imposed during the pandemic.

In addition to culture, the consequences of the COVID-19 pandemic on mental health is likely to vary between countries over time, reflecting infection rate and mortality. Qatar reported its first case of COVID-19 in late February 2020. This was followed by a rapid rise in infections, peaking in late May, before falling during June and July 2020. From August 2020, infection rate remained relatively low until February 2021 when a second wave began. The Government managed the first wave through a strict lockdown, restricting the entry of travelers to the country and making the wearing of facemasks in public and the use of a smart phone contact-tracing app compulsory. These policies, together with a well-resourced national health service, that provides free medical care to all residents and nationals, and a young population, account for the country’s relatively low COVID-19 mortality. A four-phase easing of the lockdown began in mid-June 2020 with phase 4 commencing on September 1st. Some lockdown measures were reintroduced in March 2021 and a COVID-19 vaccination programme stated in December 2020.

At the time of writing (November 2021), the total number of people tested for COVID-19 in Qatar is 2,854,019. The number of cases who tested positive is 239,752, while the number of recovered patients is 237,801. The number of active COVID-19 under treatment is 1340 and the total number of deaths is 611 [10].

We conducted the first cross-sectional online survey to identify factors associated with symptoms of depression and anxiety during the first wave of the COVID-19 pandemic in adults living in Qatar. In addition to variables that are typically associated with depression and anxiety including younger age [11], ethnicity [12], female [13], loneliness [14], and prior history of mental illness [15, 16], we investigated several COVID-19 related factors. We predicted that being infected with or having a close friend or relative infected with COVID-19 or having experienced death of a family member or friend or having been quarantined due to COVID-19 would be positively associated with depressive and anxiety symptomology. The potential COVID-19 related risk factors we chose were based on a literature review of risk factors for depression and anxiety associated with previous epidemics. Religiosity was hypothesized to be negatively associated with moderate-to-severe levels of depressive and anxiety symptoms though findings from studies conducted prior to the COVID-19 pandemic have been mixed [17, 18]. We hypothesized that worries related to use of social media outlets for COVID-19 related news and updates would be positively associated with moderate to severe levels of these symptoms as per existing findings on negative impact of social media use on mental health [19]. Any changes in living arrangements or employment status were hypothesized to be significant stressors that would be positively associated with moderate to severe levels of depression or anxiety symptoms. We report findings from data collected between July and December 2020, after the first COVID-19 wave had resolved in Qatar and loosening of lockdown measures had started.

## Methods

### Study design and sample

A convenience sample of 1,138 participants were recruited through social media and completed a cross-sectional online survey made available on the study website from July to December 2020. Participants were included in the study if they were a resident of Qatar (nationals and expatriates), age 18 years and above, and read/spoke Arabic or English.

### Ethics

Qatar University Institutional Review Board (QU-IRB 1338 EA/20) and Hamad Medical Corporation (MRC05-089) approved the study protocol in accordance with standard research protocols and HIPAA. Electronic consent was obtained from each respondent.

### Participation procedures

The study website, adverts, study information and questionnaire were available in Arabic and English. Arabic is the official language of Qatar and English is widely spoken. The survey was programmed in Qualtrics [20]. The first page of the Qualtrics survey link showed a hyperlink of the study information sheet. After participants confirmed that they have read the study information sheet, they were required to complete a tick box consent form before being able to complete the survey. The entire survey required approximately 30 min to complete.

### Translation

For our main dependent variable, officially translated versions of the PHQ-9 and GAD-7 in Arabic were obtained from the following website [21] that was developed by the MAPI research trust (https://mapi-trust.org/about-us/) using internationally accepted translation methodology [22]. Both of these instruments have been previously validated in Arabic speaking populations [40] and in large population-based studies in Qatar [23–25]. For other psychosocial indices, we used the process of translation and adaptation of instruments as outlined by the WHO guidelines [26].

### Measures

#### Depression symptoms

The nine-item Physician Health Questionnaire (PHQ-9) is a relatively brief and well-validated screening measure of depression used globally in both clinical and general population samples [27–35]. The PHQ-9 captures the frequency of nine symptom criteria for diagnosis of Major Depressive Episode (MDE) in the DSM-5 (American Psychiatric Association, 2013) within the past 2 weeks with 4-point response options for each symptom: 0 = “not at all,” 1 = “several days,” 2 = “more than half the days,” and 3 = “nearly every day.” Total scores can range from 0 to 27. In the general population, several studies have established the validity of the PHQ-9 or shorter versions [36–40]. The PHQ-9 has been validated in an Arabic-speaking outpatient population with the sensitivity and specificity for diagnosing MDE using a cutoff of 10 being 77% and 46%, respectively [40]. Item 9 of the PHQ-9 (thoughts that you would be better off dead or thoughts of hurting yourself in some way) assesses passive thoughts of death or self-injury. It is sometimes used as a screener for suicide risk. However, its predictive value has been reported to be low [41].

#### Anxiety symptoms

The GAD-7 is a relatively brief and well-validated screening measure for GAD symptoms used in both clinical and community samples to date [42]. Although not a diagnostic instrument, the GAD-7 captures the frequency of seven symptom criteria for GAD in the DSM-5 [43] over the past 2 weeks with the same 4-point response options for each symptom as in the PHQ-9. Cut-off scores of 5, 10, and 15 denote mild, moderate and severe GAD symptoms, respectively [44, 45]. Previous studies have shown that the GAD-7 exhibits good psychometric properties [46, 47] and acceptable sensitivity, but less-than-ideal specificity [40, 46].

#### Combined depression and anxiety symptoms

Our dependent variable was defined based on combining depression and anxiety into one scale known as PHQ-ADS [47]. The PHQ-ADS threshold scores of 10, 20, and 30 were previously found to indicate mild, moderate, and severe levels of depression and anxiety symptoms, respectively [47]. As per previous findings, a cut-off of 20 or higher was used in our study to dichotomize our dependent variable into moderate-to-severe levels versus mild levels or no symptoms of anxiety or depression [47].

#### Past psychiatric history

We also assessed whether the respondent had any previous history of mental illness by administering the following question: “Have you ever been diagnosed with mental health problems by a professional even if you don't have it currently? By professional we mean any doctor, nurse or person with specialized training such as a psychologist or psychiatrist”.

#### Pandemic-related questions

We included questions about personal history of COVID-19 (reported positive status confirmed by a test), death of a family member or a friend due to COVID-19, and any experience with quarantine since the pandemic started. We also asked respondents about any changes in their living arrangement since the pandemic started.

#### COVID-19 related social media use

Among those who indicated any social media use for finding COVID-19 related news or updates, we asked the following question: “Did surfing the net and/or using social media to look for coronavirus updates, alter your level of worry about coronavirus?” Respondents answered using one of three following options: “yes, it reduced my worries”, “no, it had no effect on my worries”, and “yes, it increased my worries”.

#### Loneliness

We assessed levels of loneliness using a 3-item version of the revised UCLA loneliness scale [48], which have been shown to have good convergent and discriminant validity as well as reliability in large population-based health surveys [49]. The first item was “how often do you feel that you lack companionship?” The second item was “how often do you feel left out?” and the third item was “how often do you feel isolated from others?” The following three response options were provided for each item or statement: 1 = “Hardly ever”, 2 = “Some of the time”, and 3 = “Often”. As in the revised UCLA scale, responses on these three questions were summed for each participant, with higher scores indicating greater loneliness.

#### Religiosity

We administered a brief measure of religiosity, the 5-item Duke University Religion Index or DUREL, which assess intrinsic and extrinsic (organizational and non-organizational) dimensions of religiosity [50]. First, for each of the three subscales, the items were summed into a composite score. As recommended by the authors of the scale, we then explored how each of the three subscales correlated with our dependent variable, the PHQ-ADS, and with each other, before summing all items into one index score for each respondent, with higher scores indicating greater religiosity.

#### Sociodemographic and other variables

We collected standard information about age, gender, nationality, education, marital, and employment status. Cultural background or ethnicity was determined based on questions in relation to nationality (country of origin) and language chosen to complete the interview (Arabic versus English). We used these variables to define two cultural groups: Arab versus non-Arab as we were most interested in accounting for cultural differences in depression and anxiety symptoms between mainstream culture of Qatar (Arabic) versus other cultures.

### Statistical analysis

For each scale, we assessed distributional properties (normality, skewness, and kurtosis) as well as inter-item (item-test and item-rest) correlations, Cronbach’s alpha, and average (inter-item) covariance.

We calculated descriptive statistics including proportions/percentages, mean, standard deviation (SD), and standard errors (SEs) for all variables in the study.

For bivariate associations, the Chi-square test of proportions were initially used to compare the distribution of the categorical independent variables across the two levels of the PHQ-ADS defined by a cut-off of 20 or higher: moderate-to-severe versus mild or no symptoms of depression or anxiety. Similarly, we used one way ANOVA (F-test) to compare the mean scores of loneliness and religiosity across the two-levels of PHQ-ADS.

We also fitted univariable (one variable only) and multivariable logistic regression models to identify associations between the above mentioned potential explanatory variables identified from the literature and moderate-to-severe levels relative to mild levels or no symptomology of depression or anxiety as the reference group. We estimated odds ratios (ORs) with corresponding 95% confidence intervals (CI) and robust SEs from the exponentiated coefficients of associations between these variables and our dependent variable—the PHQ-ADS.

Only one potential explanatory variable was entered into each univariable model and the corresponding unadjusted OR was estimated for the association between each variable and the PHQ-ADS. For multivariable models, we fitted a fully-adjusted model by simultaneously regressing all of the following potential explanatory variables on PHQ-ADS: Arab ethnicity, nationality (Qatari versus non-Qatari), gender, age, marital status, employment status, previous mental illness status, COVID-19 related variables (personal infection status, death or infection of someone in the immediate social circle of the respondent, quarantine status, change in living arrangements), effect of COVID-19 related social media use, loneliness, and religiosity. Another competing model was also fitted – a reduced model fitted based on adjusting for only the variables that were statistically significant in the fully-adjusted model in addition to adjustment for age and gender.

We evaluated the fit of our models using the Pearson-chi square goodness of fit test and the likelihood ratio test. The latter was carried out to compare the fit of the fully-adjusted model versus the reduced model.

To evaluate the effect of missing data on robustness of our findings, we conducted a sensitivity analysis by re-running our logistic regression models that were based on complete data analysis (list wise deletion) using full information maximum likelihood estimation (FIML) with Monte Carlo integration algorithm (2000 iterations).

All statistical analyses were carried out in Stata [51] with the exception of our sensitivity analysis, which was conducted in Mplus (Asparouhov, n.d.). A p-value less than 0.05 (typically ≤ 0.05) was considered statistically significant.

## Results

The total initial sample was 2,134. However, after restricting our analysis to include only those participants who completed at least 80% of the survey and responded to basic demographic (age and gender), our total final sample was reduced to 957 participants. Approximately, 33.0% of questionnaires were completed in English, while 67.0% of the questionnaires were completed in Arabic.

The characteristics of the respondents are shown in Table 1. The majority were female (71.0%), Arabs (69.0%), and Non-Qataris (70.0%). Approximately 52.0% were married and 51.0% were employed. The sample comprised of 28.0% in the youngest age category (18 to 24 years), while 23.0% of the sample was 40 years of age and above.

**Table 1:** Sample Characteristics and Bivariate Associations with Moderate-to-Severe Depression or Anxiety Symptoms (dependent variable)

	Frequency (n)	Percentage (%)	Frequency equal or above cut-off score of 20 on the PHQ-ADS (n)	Percentage equal or above cut-off score of 20 on the PHQ-ADS (%)	Chi/F-statistic (P-value)
Ethnicity					
Arab	658	69.0	239	37.0	0.581
Non-Arab	299	31.0	101	35.0	
Nationality					
Non-Qatari	673	70.0	236	36.0	0.750
Qatari	284	30.0	104	37.0	
Education level					
Below university	313	33.0	115	38.0	0.005
Under graduate	435	45.0	171	40.0	
Graduate	208	22.0	54	27.0	
Gender					
Male	276	29.0	71	26.0	0.000
Female	681	71.0	269	40.0	
Age group (years)					
18–24	272	28.0	132	49.0	0.000
25–29	172	18.0	77	46.0	
30–34	168	18.0	47	29.0	
35–39	123	13.0	33	27.0	
40 +	222	23.0	51	24.0	
Marital status					
Ever married	495	52.0	136	28.0	0.000
Never married	462	48.0	204	45.0	
Employment status					
Employed	488	51.0	141	29.0	0.000
Unemployed	468	49.0	199	44.0	
Social media use					
Increased worries	326	34.0	153	48.0	
No effect or reduced worries	631	66.0	187	30.0	0.000
Covid-19 illness: Self					
Yes /Maybe	131	14.0	57	44.0	
No	826	86.0	283	35.0	0.043
Covid-19 Illness: In immediate social circle					
Yes/maybe	542	57.0	199	37.0	0.456
No	414	43.0	141	35.0	
Covid-19 Family/Friend Death					
Yes	108	11.0	46	44.0	0.087
No	848	89.0	294	35.0	
Quarantine status					
Yes	135	14.0	46	35.0	0.771
No	821	86.0	294	36.0	
Previous psychiatric disorder					
Yes	159	17.0	81	53.0	0.000
No	798	83.0	259	33.0	
Covid-19 related change in living arrangement					
Yes	371	61.2	145	40.1	0.050
No	585	38.8	217	59.9	
Mean loneliness score^a^ (SD)	5.8 (2.0)	–	7.3 (1.6)	–	0.000
Mean religiosity score^b^ (SD)	26.1 (5.3)	–	25.1 (5.3)		0.000

^a^Assessed using 3-item version of the revised UCLA loneliness scale [49]

^b^Assessed using 5-item Duke University Religion Index (DUREL) [51]

In terms of COVID-related variables, 14.0% reported a positive test for COVID-19 or suspected that they contracted the virus; 14.0% reported having been quarantined, and 11.0% reported having experienced death of a family member or a friend due to COVID-19. Furthermore, 38.8% reported changes in living arrangement due to the pandemic.

The mean, standard deviation, skewness, Cronbach’s alpha, average covariance, and bivariate correlations for all continuous scales are shown in Table 2.

**Table 2:** Distribution, psychometric properties and bivariate correlations for main study scales

	Mean (SD)	Median	Range Min–Max	Skewness	Cronbach’s Alpha	Average inter-item covariance	PHQ-9	GAD-7	PHQ-ADS	Loneliness	Religiosity
PHQ-9	9.6 (7.1)	8	0–27	0.69	0.906	0.571	1.000				
GAD-7	7.4 (5.9)	6	0–21	0.74	0.922	0.664	0.800*	1.000			
PHQ-ADS	17.0 (12.4)	14	0–48	0.70	0.945	0.561	0.954*	0.932*	1.000		
Loneliness	5.8 (1.9)	6	3–9	0.10	0.815	0.362	0.585*	0.564*	0.610*	1.000	
Religiosity	26.1 (5.3)	27	11–33	− 1.15	0.776	0.887	− 0.155*	-0.168*	− 0.172*	− 0.178*	1.000

Bivariate Pearson’s correlations, N = 889 to N = 951 due to occasional missing data on paired variables. *Correlations significant at the 1.0% level after Bonferroni adjustment

The point prevalence of depressive and anxiety symptoms based on a cut-off of 10 or higher on the PHQ-9 was 43.0% (95%CI 39.9–46.2). The point prevalence of anxiety symptoms, using a cut-off of 10 or higher, on the GAD-7 was 31.6% (95%CI: 28.7–34.7). The point prevalence of moderate-to-severe depressive or anxiety symptoms on the PHQ-ADS scale, using a cut-off of 20 or higher, was 36.2% (95% CI 33.2–39.4). Based on the 9th item of the PHQ-9, approximately 24.0% of our sample reported having thoughts of death or self-injury within the last two weeks including 13.4% who reported feeling this way several days, 4.7% more than half of the days, and 6.1% nearly every day in the in the past two-weeks. The percentage of participants who reported previous history of mental health problems was 17.0%.

Results of the unvariable and multivariable models are shown in Table 3.

**Table 3:** Results from Logistic Regression Models

Variables	Univariable Models (N*)				Reduced Model (N = 887)				Fully-adjusted Model (N = 886)			
	OR	P-value	CI		OR	P-value	CI		OR	P-value	CI	
Arab ethnicity (Non-Arab ref)												
Arab	1.08	0.581	0.812	1.450	1.67	0.012	1.120	2.482	1.67	0.026	1.065	2.622
Nationality (Non-Qatari ref)												
Qatari	1.05	0.750	0.784	1.401	–	–	–	–	0.87	0.537	0.572	1.338
Education level (Diploma or Less ref)												
Graduate degree or higher	1.76	0.001	1.245	2.481	–	–	–	–	1.10	0.681	0.699	1.730
Gender (Male ref)												
Female	1.86	0.000	1.362	2.543	1.17	0.444	0.782	1.750	1.18	0.435	0.777	1.798
Age												
In years	0.96	0.000	0.949	0.975	0.99	0.216	0.968	1.007	0.99	0.283	0.968	1.009
Marital status (Never Married ref)												
Ever married	0.49	0.000	0.373	0.641	0.60	0.018	0.397	0.916	0.59	0.015	0.388	0.901
Employment status (Unemployed ref)												
Employed	0.53	0.000	0.408	0.700	–	–	–	–	0.90	0.603	0.620	1.320
Previous mental illness (No previous mental illness ref)	2.28	0.000	1.609	3.244	1.76	0.011	1.142	2.720	1.80	0.009	1.159	2.786
People in your immediate social circle infected (No ref)	1.11	0.456	0.845	1.451	–	–	–	–	0.93	0.712	0.653	1.338
Effect of social media (No effect/ reduced worries ref)												
Increased my worries	2.11	0.000	1.598	2.790	1.78	0.001	1.250	2.534	1.72	0.003	1.206	2.464
Personal History of COVID-19 (No history ref)	1.47	0.044	1.010	2.144	1.70	0.033	1.043	2.778	1.76	0.039	1.030	3.008
Death due to the coronavirus (No death ref)	0.70	0.088	0.464	1.055	–	–	–	–	0.68	0.154	0.395	1.158
Been under quarantine (No quarantine ref)	0.94	0.771	0.642	1.390	–	–	–	–	0.90	0.703	0.527	1.540
Change in living arrangement (No change ref)	1.31	0.050	0.999	1.723	–	–	–	–	1.28	0.183	0.891	1.832
Loneliness	2.01	0.000	1.829	2.211	1.93	0.000	1.744	2.133	1.91	0.000	1.729	2.120
Religiosity	0.95	0.000	0.923	0.971	0.97	0.055	0.935	1.001	0.96	0.039	0.931	0.998

Ref, reference group; OR, Odds Ratio; CI, 95% Confidence Intervals. Note. Dependent variable is moderate to severe levels of depression or anxiety. *N varies for univariable models

Variables that were significantly associated with moderate-to-severe levels of depression or anxiety on the PHQ-ADS in univariable models were: higher educational status, female gender, younger age, never married, unemployed, prior mental illness, those who stated that social media use for COVID-19 related updates/news increased their worries, those who were infected/or suspected of being infected with COVID-19, those with higher levels of loneliness, and lower levels of religiosity (Table 3).

In the fully-adjusted model, the following variables were significantly associated with moderate-to-severe levels of depression or anxiety on the PHQ-ADS: Arab ethnicity (OR = 1.67, p = 0.026), participants who were never married versus married (OR = 2.04, p < 0.001), prior history of psychiatric disorder versus no history (OR = 1.80, p = 0.009), participants who stated that social media use for COVID-related news/updates increased their worries (OR = 1.72, p = 0.003), reported infected/or suspected of being with COVID-19 (OR = 1.76, p = 0.039), experienced higher levels of loneliness (OR = 1.91, p < 0.001), and reported lower levels of religiosity (OR = 0.96, p = 0.039) (Table 3).

Results from the reduced model (Table 3) were the same as the fully-adjusted model with the exception of religiosity, which was only marginally statistically significant (OR = 0.97, p = 0.055). Comparing the fit of these two models, the likelihood ratio statistic was 7.00 and the associated p-value was 0.428 suggesting that the reduced model did not show a statistically significant improvement in model fit compared to the fully-adjusted model. Additionally, the p-value of goodness of fit test of the fully adjusted model (Pearson χ^2^ = 867.82, p = 0.495) was greater than that of the reduced model (Pearson χ^2^ = 860.82, p = 0.476) suggesting that the former is a better fit to the data.

The results of the sensitivity analysis for these models are presented in Appendix S1 showing that these model estimates were robust in relation to item missingness in our data.

## Discussion

To our knowledge, this is the first study to report on depression and anxiety in the general population in Qatar between July and December 2020, after the first wave had resolved in Qatar, and before the second wave had started.

Our finding that a history of confirmed or suspected COVID-19 was associated with depression-anxiety resonates with findings from several studies in which a diagnosis of COVID-19 was associated with chronic or deteriorating mental health [6, 52]. To what extend this reflects psychosocial consequences (e.g. financial repercussions) versus biological consequences of infection is unclear. Neuro-inflammation is one possible biological mechanism by which COVID-19 infection could lead to depression-anxiety.

In our study, past psychiatric history was independently associated with current depression-anxiety. This is not surprising as both conditions are often recurrent. Past psychiatric history has emerged as a risk factor for anxiety and depression during the COVID-19 pandemic in many studies including two studies from the Arab world conducted in April and/or May 2020, one from the UAE [53] and the other from Saudi Arabia [54]. The data highlights the importance of maintaining mental health services for those with mental health disorders during the pandemic. In practice, services were disrupted in many countries including shortages of medication and cancelled face to face consultations. Qatar mitigated against this by setting up the first national mental health line early on during the pandemic.

We found that lower levels of religiosity were associated with depression-anxiety. A meta-analysis found that positive religious coping is associated with better mental health outcomes in those facing stressful life events [55]. Studies in various countries and communities in the early stages of the pandemic found that positive religious coping was associated with lower depression scores. This relationship was seen in Muslims (but not Christians) in a study of Arabs living in Saudi Arabia [56] and two studies from Qatar, one conducted in an elderly population being quarantined for COVID-19, [57, 58]. These findings suggest that health care professionals should assess spirituality as it may be another facet to help build resilience at times of stress.

The finding that loneliness was associated with depression-anxiety in our study is not surprising. Studies in both the US and UK have reported high rates of loneliness during the pandemic [59, 60]. Across several studies, female gender has been associated with loneliness during the pandemic in addition to younger age, lower socioeconomic status, preexisting mental health condition and living alone. There is an extensive literature showing that loneliness is associated with an increased risk of a range of psychiatric disorders [61]. The pandemic is likely to have caused loneliness through quarantine and lockdown restrictions including home working and social distancing. Several other countries, including in the UK, Poland and the US, have reported an association between loneliness and anxiety and/or depression during the pandemic [62–64]. It should be noted that our methodology assessed loneliness and depression-anxiety in the last 2 weeks during a time when infection rates had fallen after the first wave of COVID-19 and lockdown measures had been significantly relaxed. An even stronger relationship between loneliness and mood disorders may have been observed if the data were gathered when infection rates were higher and lockdown measures were more stringent. Unfortunately, no pre-pandemic data exist on the association of loneliness with depression-anxiety at the population level in Qatar. Therefore, the extent that this finding is unique to the context of COVID-19 is unknown.

Our findings in relation to female gender and Arab ethnicity being significantly associated with depression-anxiety during the pandemic is consistent with findings from population-based studies conducted in Qatar prior to the pandemic [23, 25]. We found that marriage was a significant protective factor against experience depression/anxiety. This is in keeping with most existing literature conducted both before and during the pandemic. Marital status is a well-recognized predictor of mental health in pre-pandemic studies [65]. Possible explanations include marriage providing an increased sense of purpose and structure to an individual’s life and insulating against financial hardship if the couple pool two incomes. In addition to these general benefits, marriage may also mitigate against the effect of isolation associated with working from home during COVID-19 lockdowns. A recent study from the US found that people who are married experienced better mental health during the COVID-19 pandemic than those who are unmarried [66]. Marriage emerged as a protective factor against depression in a study conducted in Palestine in April 2020 at which time the country was under a COVID-19 lockdown [67]. Surprisingly a study from Kuwait reported that marriage was associated with depression during the COVID-19 pandemic [68]. It is also important to highlight that the relationship between marriage and mental health will vary at an individual level. COVID-19 pandemic related stressors, including increased proximity within the home, can precipitate or exacerbate family violence, which may explain some of the inconsistent findings in the literature. Social distancing may also reduce the ability of victims of abuse to support and help. Many countries have seen increased reports of domestic violence during the COVID-19 pandemic [69]. In Qatar, being married did not appear to confer protection against depression-anxiety in population-based studies that used the same instruments prior to the pandemic [23, 25]. This contrasts with our current finding and may suggest that marriage may indeed be a unique protective factor for depression-anxiety in Qatar during the pandemic.

Participants who reported that using social media use for finding COVID-19 related news/updates had increased their level of worry, versus not changing it or reducing it, were more likely to experience anxiety/depression. Internet use has been associated with anxiety/depression in several studies conducted during the pandemic [19, 70]. This includes a study conducted across six Arab countries (Oman, Saudi Arabia, Jordan, Iraq, United Arab Emirates, and Egypt) in April 2021 in which internet use was an independent predictor of stress, anxiety and depression in linear regression models [70].

Younger age was significantly associated with anxiety/depression in the unadjusted, but not in the reduced and fully adjusted models of our data. This could be due to confounding by age-related factors (such as employment and worries-related to social media use), which were controlled for in the adjusted models. For Qatar, population-based studies conducted prior to the pandemic also  support the finding that younger age is associated with depression-anxiety [23, 25]. However, none of these studies controlled for variables like excessive social media use that maybe associated with a younger cohort and poor psychological health, which may account for the discrepancy in this finding between pre- and post- pandemic data.

Infection with COVID-19 of people in the immediate social circle (e.g. a friend or a family relative) of the respondent did not emerge as a significant associate of depression/anxiety in our study. In contrast knowing a person who had COVID-19 was associated with higher rates of anxiety/depression in two studies from the Arab world in the early stage of the pandemic in spring 2020 [56]. The difference may reflect the timing of these studies. Our study gathered data between July and December 2020 whereas the two other studies were conducted in April/May 2020 when public knowledge of COVID-19 was less and there may have been greater concerns of contracting COVID-19 and the impact on personal health.

Various interventions may be considered for combatting pandemic associated loneliness including connecting to friends and relatives by telephone and social media, using online group games, and the use of support groups. However, careful use of social media is required as shown by our finding that worries related to excessive searching for COVID-19 related information was associated with depression and/or anxiety. This finding resonates with other research. A study in China found that spending ≥ 2 h daily on COVID-19 news via social media was associated with probable anxiety and depression in community-based adults [19].

## Limitations

The study has several limitations. A major weakness is the convenience nature of the sample. As such, it is not meaningful to comment on the prevalence of either depression or anxiety. Nevertheless, the data can be used to investigate factors associated with depression and/or anxiety. A further limitation is that the data is cross-sectional, which means that causal and temporal inferences cannot be made. We investigated several COVID-19 related factors as associates of anxiety/depression including being infected with or having a close friend or relative infected with COVID-19, experiencing death of a family member or friend or having been quarantined due to COVID-19. Since we conducted our study, a comprehensive validated scale for COVID-19 as complex traumatic stress has been developed in the Arab World [71, 72]. This 12-item scale includes three subscales assessing threat/fear of infection and death, traumatic economic stress, isolation, and disturbed routines. This scale should benefit future research in the Arab region. Finally, we did not conduct invariance analysis to confirm that those participants completing the study in English and Arabic understood concepts in the same way. This weakness needs to be balanced against the fact that the Arabic versions of two scales that formed the outcome (GAD-7 and PHQ-9) have previously been validated in non-Arab and Arab countries [40]. COVID-19 has been conceptualized as a new type of traumatic stress and it is clear that it can impact mental health in various ways not only in the general population, but also in those who are infected [73]. Our study only assessed the potential impact on depressive and anxiety symptoms due to the need to keep our questionnaire short to facilitate a high response rate. It is well recognized that the COVID-19 pandemic has increased the prevalence of symptoms of posttraumatic stress disorder and it is likely there may be effects on other psychiatric disorders including insomnia, obsessive–compulsive disorder, and panic disorder. Future studies should assess these domains.

In terms of strengths, we employed a range of standardized instruments including the GAD-7 and PHQ-9 to assess depression and anxiety. In contrast, some studies have assessed anxiety and depression in the general population during the pandemic using single-item questions [74]. Where possible we used validated Arabic translations of the instruments. Our tool was available nationwide via social media in two languages making it relatively easy for people to participate. We achieved a reasonable sample size (analyzable data for 957 participants).

## Conclusions

This study adds to the growing literature on potential risk factors for depression and anxiety symptoms during the pandemic. The factors we identified may assist in designing support and interventions for those at greater risk of depression and anxiety in future COVID-19 waves or lockdowns in Qatar and other Arab-speaking countries. The data presented here represent findings at one point during the pandemic in Qatar. The pandemic is a fluid chronic event and further work is needed to determine how psychiatric morbidity varies at different points in the pandemic and between countries.

## Supplementary Information

Supplementary file 1. (DOCX 24 KB)

## Data Availability

The data that support the findings of this study are available from the corresponding author upon reasonable request and pending additional ethical approval.

## Abbreviations

ANOVA: Analysis of Variance
COVID-19: Coronavirus disease 2019
GAD-7: 7-Item Generalized Anxiety Disorder
PHQ-9: The 9-item Patient Health Questionnaire
PHQ-ADS: Patient Health Questionnaire and Anxiety Depression Scale
UCLA: University of California, Los Angeles
WHO: World Health Organization
