# Supplementary material for: Factors associated with depression and anxiety in the adult population of Qatar after the first COVID-19 wave: a cross-sectional study
Source: Discov Psychol. 2021 Dec 20;1(1):9. doi: 10.1007/s44202-021-00009-z (PMC8686347; doi:10.1007/s44202-021-00009-z)
Supplement: Supplementary file 1 — (DOCX 24 KB) [file 44202_2021_9_MOESM1_ESM.docx]

Appendix S1: Logistic Regression Models using Full Information Maximum Likelihood instead of Listwise deletion

|  | **Reduced Model** (N=957) | | **Fully Adjusted Model** (N=957) | |
| --- | --- | --- | --- | --- |
| **Variables** | OR | P-value | OR | P-value |
| Arab ethnicity (Non-Arab ref) |  |  |  |  |
| Arab | 1.71 | 0.007 | 1.73 | 0.015 |
| Nationality (Non-Qatari ref) |  |  |  |  |
| Qatari | --- | --- | 0.93 | 0.716 |
| Education Level (Diploma or Less ref) |  |  |  |  |
| Graduate degree or higher | --- | --- | 1.15 | 0.529 |
| Gender (Male ref) |  |  |  |  |
| Female | 1.23 | 0.310 | 1.25 | 0.283 |
| Age in years |  |  |  |  |
| 1-year increase in Age | 0.98 | 0.127 | 0.99 | 0.171 |
| Marital Status (Never Married ref) |  |  |  |  |
| Ever married | 0.61 | 0.016 | 0.61 | 0.018 |
| Employment Status (Unemployed ref) ­ |  |  |  |  |
| Employed | --- | --- | 0.95 | 0.781 |
| Previous Mental Illness (No ref) |  |  |  |  |
| Yes | 1.77 | 0.009 | 1.80 | 0.008 |
| People in Immediate Social Circle Infected (No ref) |  |  |  |  |
| Yes | --- | --- | 0.93 | 0.699 |
| Effect of Social Media (No effect/ reduced worries ref) |  |  |  |  |
| Increased worries | 1.92 | 0.000 | 1.90 | 0.000 |
| COVID-19 Infection Status (No ref) |  |  |  |  |
| Yes | 1.71 | 0.028 | 1.80 | 0.028 |
| Death of Family/Friend due COVID-19 (No ref) |  |  |  |  |
| Yes | --- | --- | 0.64 | 0.094 |
| Have Been Quarantined (No ref) |  |  | --- | --- |
| Yes | --- | --- | 0.86 | 0.571 |
| Change in Living Arrangement (No ref) |  |  |  |  |
| Yes | --- | --- | 1.18 | 0.353 |
| Loneliness |  |  |  |  |
| 1-unit increase in loneliness score | 1.94 | 0.000 | 1.93 | 0.000 |
| Religiosity |  |  |  |  |
| 1-unit increase in religiosity score | 0.97 | 0.050 | 0.96 | 0.036 |

Abbreviations: OR, Odds Ratio; CI, 95% Confidence Intervals. Note. Dependent variable is moderate to severe levels of depression and anxiety.
